# 5,8-Dimeth­oxy-3,9-dimethyl-3a,4,9,9a-tetra­hydro-4,9-ep­oxy­naphtho­[2,3-*d*]isoxazole

**DOI:** 10.1107/S1600536814007752

**Published:** 2014-04-12

**Authors:** Alan J. Lough, Jaipal R. Nagireddy, William Tam

**Affiliations:** a Department of Chemistry, University of Toronto, Toronto, Ontario, M5S 3H6, Canada; b Department of Chemistry, University of Guelph, Guelph, Ontario, N1G 2W1, Canada

## Abstract

The title compound, C_15_H_17_NO_4_, is the *exo* isomer with a *syn* arrangement of the O atom in the isoxazole ring to the methyl group of the bicyclic alkene. The dihedral angle between the isoxazole ring and the benzene ring is 7.42 (9)°. In the crystal, weak C—H⋯O hydrogen bonds link mol­ecules, forming a three-dimensional network. The isoxazole O atom is an acceptor for both weak hydrogen bonds.

## Related literature   

For 1,3-dipolar cyclo­addition reactions of symmetrical and unsymmetrical bicyclic alkenes, see: Yip *et al.* (2001 ▶); Mayo *et al.* (2001 ▶). For a related structure, see: Lough *et al.* (2014 ▶).
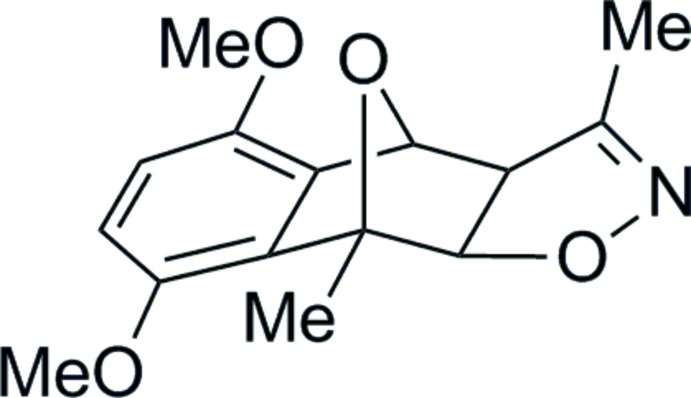


## Experimental   

### 

#### Crystal data  

C_15_H_17_NO_4_
*M* *_r_* = 275.29
Monoclinic, 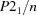

*a* = 9.0608 (12) Å
*b* = 14.3998 (17) Å
*c* = 10.1631 (12) Å
β = 104.835 (3)°
*V* = 1281.8 (3) Å^3^
*Z* = 4
Mo *K*α radiation
μ = 0.10 mm^−1^
*T* = 147 K
0.32 × 0.16 × 0.14 mm

#### Data collection  

Bruker Kappa APEX DUO CCD diffractometer
Absorption correction: multi-scan (*SADABS*; Bruker, 2012 ▶) *T*_min_ = 0.660, *T*_max_ = 0.746
11844 measured reflections
2937 independent reflections
2271 reflections with *I* > 2σ(*I*)
*R*_int_ = 0.041

#### Refinement  

*R*[*F* ^2^ > 2σ(*F* ^2^)] = 0.039
*wR*(*F* ^2^) = 0.097
*S* = 1.05
2937 reflections
185 parameters
H-atom parameters constrained
Δρ_max_ = 0.28 e Å^−3^
Δρ_min_ = −0.22 e Å^−3^

### 

Data collection: *APEX2* (Bruker, 2012 ▶); cell refinement: *SAINT* (Bruker, 2012 ▶); data reduction: *SAINT*; program(s) used to solve structure: *SHELXS97* (Sheldrick, 2008 ▶); program(s) used to refine structure: *SHELXL2013* (Sheldrick, 2008 ▶); molecular graphics: *PLATON* (Spek, 2009 ▶); software used to prepare material for publication: *SHELXTL* (Sheldrick, 2008 ▶).

## Supplementary Material

Crystal structure: contains datablock(s) I. DOI: 10.1107/S1600536814007752/is5351sup1.cif

Structure factors: contains datablock(s) I. DOI: 10.1107/S1600536814007752/is5351Isup2.hkl

Supporting information file. DOI: 10.1107/S1600536814007752/is5351Isup3.cml

CCDC reference: 995953

Additional supporting information:  crystallographic information; 3D view; checkCIF report

## Figures and Tables

**Table 1:** Hydrogen-bond geometry (Å, °)

*D*—H⋯*A*	*D*—H	H⋯*A*	*D*⋯*A*	*D*—H⋯*A*
C1—H1*A*⋯O2^i^	1.00	2.35	3.2928 (17)	156
C14—H14*C*⋯O2^ii^	0.98	2.59	3.5340 (19)	161

Symmetry codes: (i) 
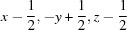
; (ii) 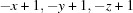.

**Fig. 1.**
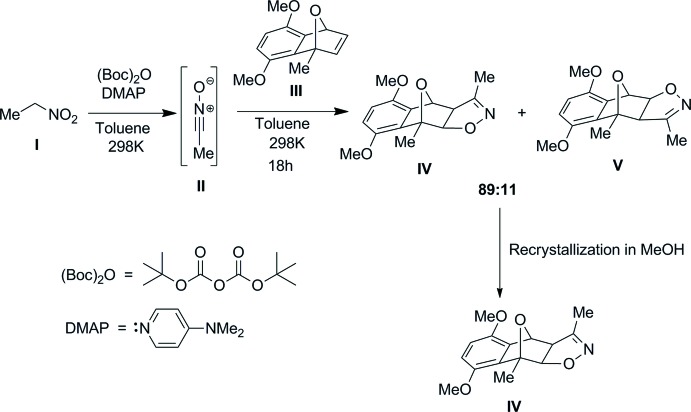
The reaction scheme.

**Fig. 2.**
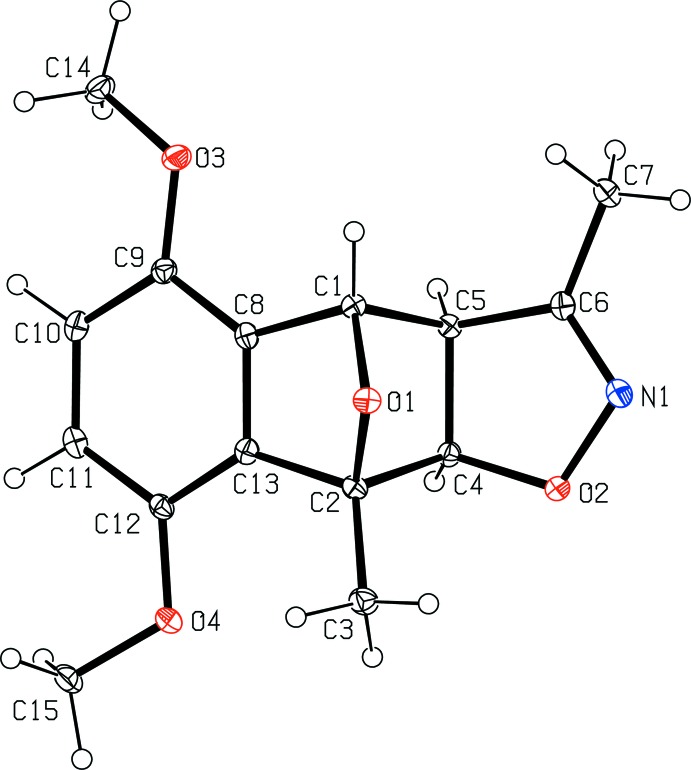
The molecular structure of the title compound showing 30% probability ellipsoids.

**Fig. 3.**
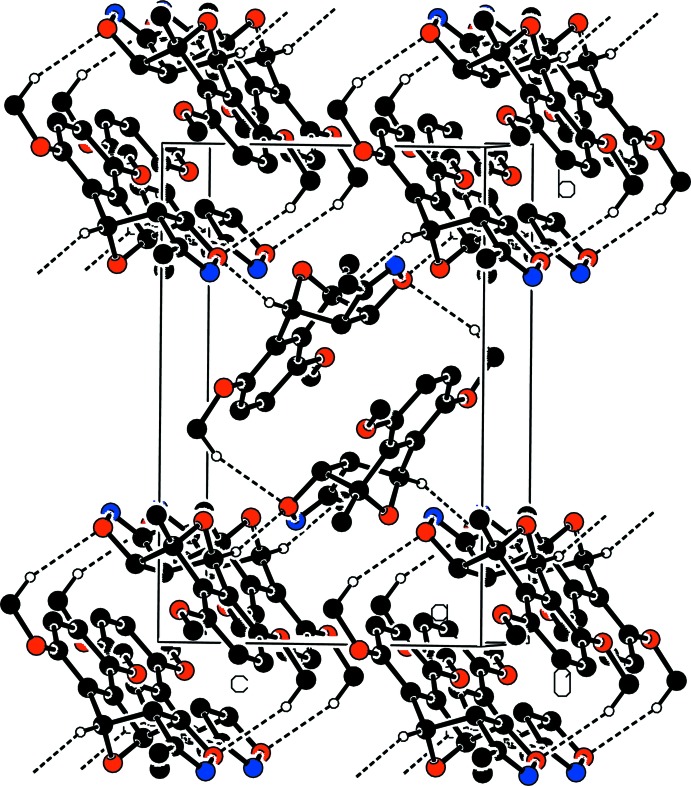
Part of the crystal structure with weak hydrogen bonds shown as dashed lines.

## supplementary crystallographic information

### 1. Comment

We have previously investigated the 1,3-dipolar cycloaddition reactions of symmetrical and unsymmetrical bicyclic alkenes (Yip *et al.*, 2001; Mayo *et al.*, 2001). When expanding this reaction with C1-substituted oxabenzonorbornadienes, the bicyclic alkene (III) reacts (see Fig. 1) with acetonitrile oxide (II) (generated *in situ*) in toluene, to give the cycloadducts (IV) and (V) as regioisomers in the ratio of 89:11 respectively (ratio was determined by ^1^H NMR). The stereochemistry and regiochemistry of the major product (IV) was determined by this single-crystal X-ray analysis. Although different stereoisomers (*exo* and *endo*) could be formed, only the *exo* stereoisomer was formed with a mixture of the corresponding regioisomers. The major product (IV) obtained was found to be the product with the oxygen of the nitrile oxide *syn* to the C1-methyl group of the bicyclic alkene.

The molecular structure of the title compound is shown in Fig. 2. The dihedral angle between the isoxazole ring (C4/C5/C6/O2/N1 with an r.m.s. deviation 0.0125 Å) and the benzene ring (C8–C13) is 7.42 (9)°. In the crystal, weak C—H···O hydrogen bonds link molecules forming a three-dimensional network (Fig. 3). The isoxazole O atom is an acceptor for both weak hydrogen bonds. We have prepared by a similar method and carried out the structure determination of a related cycloadduct (Lough *et al.*, 2014)

### 2. Experimental

A solution of nitroethane (I) (130.0 mg, 1.733 mmol) in toluene (2 ml) was added to a flame-dried flask containing bicyclic alkene (II) (140 mg, 0.642 mmol), (BOC)_2_O (233.7 mg, 1.07 mmol), DMAP (9.4 mg, 0.077 mmol) and toluene (6 ml) *via* a cannula over 10 minutes. The reaction mixture was stirred at room temperature for 18 h. The solvent was removed by rotary evaporation, and the crude product was purified by column chromatography (EtOAc:hexanes = 1:9 to 8:2) followed by recrystallization in methanol to give cycloadduct (IV) in 70% yield. Recrystallization of a solution of the title compound in MeOH provided crystals suitable for X-ray diffraction.

### 3. Refinement

Hydrogen atoms were placed in calculated positions with C—H distances of 0.95–1.00 Å and included in the refinement in a riding-model approximation with *U*_iso_(H) = 1.2*U*_eq_(C) or 1.5*U*_eq_(C_methyl_).

### Crystal data

**Table:**

C_15_H_17_NO_4_	*F*(000) = 584
*M**_r_* = 275.29	*D*_x_ = 1.427 Mg m^−^^3^
Monoclinic, *P*2_1_/*n*	Mo *K*α radiation, λ = 0.71073 Å
*a* = 9.0608 (12) Å	Cell parameters from 2761 reflections
*b* = 14.3998 (17) Å	θ = 2.5–27.5°
*c* = 10.1631 (12) Å	µ = 0.10 mm^−^^1^
β = 104.835 (3)°	*T* = 147 K
*V* = 1281.8 (3) Å^3^	Needle, colourless
*Z* = 4	0.32 × 0.16 × 0.14 mm

### Data collection

**Table:**

Bruker Kappa APEX DUO CCD diffractometer	2271 reflections with *I* > 2σ(*I*)
Radiation source: sealed tube with Bruker Triumph monochromator	*R*_int_ = 0.041
φ and ω scans	θ_max_ = 27.5°, θ_min_ = 2.5°
Absorption correction: multi-scan (*SADABS*; Bruker, 2012)	*h* = −11→11
*T*_min_ = 0.660, *T*_max_ = 0.746	*k* = −18→17
11844 measured reflections	*l* = −11→13
2937 independent reflections	

### Refinement

**Table:**

Refinement on *F*^2^	0 restraints
Least-squares matrix: full	Hydrogen site location: inferred from neighbouring sites
*R*[*F*^2^ > 2σ(*F*^2^)] = 0.039	H-atom parameters constrained
*wR*(*F*^2^) = 0.097	*w* = 1/[σ^2^(*F*_o_^2^) + (0.0459*P*)^2^ + 0.2574*P*] where *P* = (*F*_o_^2^ + 2*F*_c_^2^)/3
*S* = 1.05	(Δ/σ)_max_ < 0.001
2937 reflections	Δρ_max_ = 0.28 e Å^−^^3^
185 parameters	Δρ_min_ = −0.22 e Å^−^^3^

### Special details

**Table:**

Geometry. All e.s.d.'s (except the e.s.d. in the dihedral angle between two l.s. planes) are estimated using the full covariance matrix. The cell e.s.d.'s are taken into account individually in the estimation of e.s.d.'s in distances, angles and torsion angles; correlations between e.s.d.'s in cell parameters are only used when they are defined by crystal symmetry. An approximate (isotropic) treatment of cell e.s.d.'s is used for estimating e.s.d.'s involving l.s. planes.

### Fractional atomic coordinates and isotropic or equivalent isotropic displacement parameters (Å^2^)

**Table:**

	*x*	*y*	*z*	*U*_iso_*/*U*_eq_	
O1	0.50314 (11)	0.25637 (7)	0.36251 (9)	0.0158 (2)	
O2	0.59142 (11)	0.27152 (7)	0.66027 (10)	0.0199 (2)	
O3	0.27204 (11)	0.49102 (7)	0.15675 (10)	0.0217 (2)	
O4	0.89378 (11)	0.43196 (7)	0.37701 (10)	0.0205 (2)	
N1	0.44029 (14)	0.24274 (9)	0.65909 (12)	0.0200 (3)	
C1	0.39743 (15)	0.33293 (10)	0.34640 (13)	0.0147 (3)	
H1A	0.2909	0.3190	0.2927	0.018*	
C2	0.63942 (15)	0.30606 (9)	0.43544 (13)	0.0147 (3)	
C3	0.77661 (17)	0.24375 (10)	0.46760 (15)	0.0201 (3)	
H3A	0.8081	0.2309	0.3841	0.030*	
H3B	0.7510	0.1853	0.5060	0.030*	
H3C	0.8603	0.2743	0.5338	0.030*	
C4	0.58557 (15)	0.34340 (10)	0.55933 (13)	0.0148 (3)	
H4A	0.6418	0.4007	0.5992	0.018*	
C5	0.41454 (15)	0.36162 (10)	0.49764 (13)	0.0141 (3)	
H5A	0.3833	0.4272	0.5086	0.017*	
C6	0.34470 (16)	0.29242 (10)	0.57416 (14)	0.0162 (3)	
C7	0.17703 (16)	0.27995 (11)	0.55319 (15)	0.0203 (3)	
H7A	0.1580	0.2282	0.6092	0.030*	
H7B	0.1300	0.2665	0.4570	0.030*	
H7C	0.1328	0.3369	0.5795	0.030*	
C8	0.47998 (16)	0.40647 (10)	0.28645 (13)	0.0145 (3)	
C9	0.42749 (16)	0.48105 (10)	0.20091 (14)	0.0163 (3)	
C10	0.53678 (17)	0.53792 (10)	0.16776 (14)	0.0178 (3)	
H10A	0.5052	0.5885	0.1070	0.021*	
C11	0.69228 (17)	0.52157 (10)	0.22265 (14)	0.0182 (3)	
H11A	0.7647	0.5609	0.1976	0.022*	
C12	0.74383 (16)	0.44869 (10)	0.31354 (13)	0.0153 (3)	
C13	0.63422 (15)	0.39029 (9)	0.34279 (13)	0.0144 (3)	
C14	0.21634 (18)	0.57380 (10)	0.08270 (15)	0.0220 (3)	
H14A	0.1046	0.5751	0.0622	0.033*	
H14B	0.2481	0.5748	−0.0025	0.033*	
H14C	0.2581	0.6282	0.1376	0.033*	
C15	1.00530 (17)	0.47219 (11)	0.31725 (16)	0.0227 (3)	
H15A	1.1070	0.4499	0.3652	0.034*	
H15B	1.0019	0.5400	0.3244	0.034*	
H15C	0.9835	0.4543	0.2212	0.034*	

### Atomic displacement parameters (Å^2^)

**Table:**

	*U*^11^	*U*^22^	*U*^33^	*U*^12^	*U*^13^	*U*^23^
O1	0.0159 (5)	0.0130 (5)	0.0180 (5)	−0.0010 (4)	0.0035 (4)	−0.0021 (4)
O2	0.0152 (5)	0.0255 (6)	0.0188 (5)	0.0024 (4)	0.0042 (4)	0.0073 (4)
O3	0.0169 (5)	0.0212 (6)	0.0255 (5)	0.0018 (4)	0.0029 (4)	0.0078 (4)
O4	0.0145 (5)	0.0259 (6)	0.0217 (5)	−0.0024 (4)	0.0057 (4)	0.0032 (4)
N1	0.0188 (6)	0.0216 (7)	0.0211 (6)	−0.0009 (5)	0.0077 (5)	0.0020 (5)
C1	0.0149 (7)	0.0139 (7)	0.0149 (6)	−0.0003 (5)	0.0031 (5)	−0.0003 (5)
C2	0.0140 (7)	0.0143 (7)	0.0161 (6)	−0.0012 (5)	0.0044 (5)	−0.0019 (5)
C3	0.0192 (8)	0.0183 (7)	0.0240 (7)	0.0035 (6)	0.0077 (6)	0.0010 (6)
C4	0.0161 (7)	0.0142 (7)	0.0144 (6)	0.0005 (5)	0.0043 (5)	0.0010 (5)
C5	0.0140 (7)	0.0133 (7)	0.0153 (6)	−0.0003 (5)	0.0044 (5)	−0.0005 (5)
C6	0.0197 (7)	0.0140 (7)	0.0159 (6)	−0.0006 (6)	0.0065 (5)	−0.0013 (5)
C7	0.0180 (8)	0.0220 (8)	0.0228 (7)	−0.0016 (6)	0.0088 (6)	0.0004 (6)
C8	0.0171 (7)	0.0139 (7)	0.0135 (6)	−0.0013 (5)	0.0055 (5)	−0.0025 (5)
C9	0.0171 (7)	0.0177 (7)	0.0140 (6)	0.0011 (6)	0.0035 (5)	−0.0008 (5)
C10	0.0241 (8)	0.0148 (7)	0.0149 (6)	0.0005 (6)	0.0058 (6)	0.0009 (5)
C11	0.0219 (8)	0.0173 (7)	0.0176 (7)	−0.0046 (6)	0.0092 (6)	−0.0027 (6)
C12	0.0151 (7)	0.0175 (7)	0.0144 (6)	−0.0007 (6)	0.0060 (5)	−0.0031 (5)
C13	0.0178 (7)	0.0133 (7)	0.0127 (6)	0.0007 (5)	0.0048 (5)	−0.0026 (5)
C14	0.0232 (8)	0.0204 (8)	0.0209 (7)	0.0060 (6)	0.0029 (6)	0.0048 (6)
C15	0.0165 (7)	0.0279 (8)	0.0256 (8)	−0.0038 (6)	0.0091 (6)	0.0005 (6)

### Geometric parameters (Å, º)

**Table:**

O1—C1	1.4420 (16)	C5—C6	1.5001 (19)
O1—C2	1.4550 (16)	C5—H5A	1.0000
O2—N1	1.4278 (16)	C6—C7	1.490 (2)
O2—C4	1.4487 (16)	C7—H7A	0.9800
O3—C9	1.3719 (17)	C7—H7B	0.9800
O3—C14	1.4309 (17)	C7—H7C	0.9800
O4—C12	1.3680 (17)	C8—C9	1.387 (2)
O4—C15	1.4282 (17)	C8—C13	1.3877 (19)
N1—C6	1.2744 (19)	C9—C10	1.391 (2)
C1—C8	1.5118 (18)	C10—C11	1.396 (2)
C1—C5	1.5605 (18)	C10—H10A	0.9500
C1—H1A	1.0000	C11—C12	1.397 (2)
C2—C3	1.4997 (19)	C11—H11A	0.9500
C2—C13	1.5288 (19)	C12—C13	1.3903 (19)
C2—C4	1.5580 (18)	C14—H14A	0.9800
C3—H3A	0.9800	C14—H14B	0.9800
C3—H3B	0.9800	C14—H14C	0.9800
C3—H3C	0.9800	C15—H15A	0.9800
C4—C5	1.5381 (18)	C15—H15B	0.9800
C4—H4A	1.0000	C15—H15C	0.9800
			
C1—O1—C2	97.72 (10)	C7—C6—C5	123.78 (12)
N1—O2—C4	109.89 (10)	C6—C7—H7A	109.5
C9—O3—C14	116.97 (11)	C6—C7—H7B	109.5
C12—O4—C15	116.97 (11)	H7A—C7—H7B	109.5
C6—N1—O2	109.07 (11)	C6—C7—H7C	109.5
O1—C1—C8	101.45 (10)	H7A—C7—H7C	109.5
O1—C1—C5	101.24 (10)	H7B—C7—H7C	109.5
C8—C1—C5	106.07 (11)	C9—C8—C13	122.42 (13)
O1—C1—H1A	115.4	C9—C8—C1	132.02 (13)
C8—C1—H1A	115.4	C13—C8—C1	105.43 (12)
C5—C1—H1A	115.4	O3—C9—C8	116.40 (12)
O1—C2—C3	111.39 (11)	O3—C9—C10	126.44 (13)
O1—C2—C13	100.84 (10)	C8—C9—C10	117.16 (13)
C3—C2—C13	120.13 (12)	C9—C10—C11	120.80 (13)
O1—C2—C4	100.42 (10)	C9—C10—H10A	119.6
C3—C2—C4	116.36 (11)	C11—C10—H10A	119.6
C13—C2—C4	104.91 (11)	C10—C11—C12	121.55 (13)
C2—C3—H3A	109.5	C10—C11—H11A	119.2
C2—C3—H3B	109.5	C12—C11—H11A	119.2
H3A—C3—H3B	109.5	O4—C12—C13	118.06 (12)
C2—C3—H3C	109.5	O4—C12—C11	124.58 (13)
H3A—C3—H3C	109.5	C13—C12—C11	117.35 (13)
H3B—C3—H3C	109.5	C8—C13—C12	120.62 (13)
O2—C4—C5	105.12 (11)	C8—C13—C2	104.85 (12)
O2—C4—C2	111.31 (11)	C12—C13—C2	134.47 (13)
C5—C4—C2	102.75 (10)	O3—C14—H14A	109.5
O2—C4—H4A	112.3	O3—C14—H14B	109.5
C5—C4—H4A	112.3	H14A—C14—H14B	109.5
C2—C4—H4A	112.3	O3—C14—H14C	109.5
C6—C5—C4	100.96 (11)	H14A—C14—H14C	109.5
C6—C5—C1	112.71 (11)	H14B—C14—H14C	109.5
C4—C5—C1	101.07 (11)	O4—C15—H15A	109.5
C6—C5—H5A	113.6	O4—C15—H15B	109.5
C4—C5—H5A	113.6	H15A—C15—H15B	109.5
C1—C5—H5A	113.6	O4—C15—H15C	109.5
N1—C6—C7	121.36 (13)	H15A—C15—H15C	109.5
N1—C6—C5	114.85 (13)	H15B—C15—H15C	109.5
			
C4—O2—N1—C6	−3.33 (15)	O1—C1—C8—C13	−32.41 (13)
C2—O1—C1—C8	51.19 (11)	C5—C1—C8—C13	72.97 (13)
C2—O1—C1—C5	−57.97 (11)	C14—O3—C9—C8	172.37 (12)
C1—O1—C2—C3	−179.30 (11)	C14—O3—C9—C10	−8.0 (2)
C1—O1—C2—C13	−50.69 (11)	C13—C8—C9—O3	−177.44 (12)
C1—O1—C2—C4	56.86 (11)	C1—C8—C9—O3	−2.2 (2)
N1—O2—C4—C5	2.76 (13)	C13—C8—C9—C10	2.9 (2)
N1—O2—C4—C2	−107.78 (12)	C1—C8—C9—C10	178.11 (13)
O1—C2—C4—O2	78.42 (12)	O3—C9—C10—C11	178.30 (13)
C3—C2—C4—O2	−41.92 (16)	C8—C9—C10—C11	−2.1 (2)
C13—C2—C4—O2	−177.29 (11)	C9—C10—C11—C12	−0.8 (2)
O1—C2—C4—C5	−33.63 (12)	C15—O4—C12—C13	161.27 (12)
C3—C2—C4—C5	−153.97 (12)	C15—O4—C12—C11	−19.88 (19)
C13—C2—C4—C5	70.66 (12)	C10—C11—C12—O4	−176.08 (12)
O2—C4—C5—C6	−1.30 (13)	C10—C11—C12—C13	2.8 (2)
C2—C4—C5—C6	115.27 (11)	C9—C8—C13—C12	−0.9 (2)
O2—C4—C5—C1	−117.31 (11)	C1—C8—C13—C12	−177.18 (12)
C2—C4—C5—C1	−0.74 (13)	C9—C8—C13—C2	176.63 (12)
O1—C1—C5—C6	−71.58 (13)	C1—C8—C13—C2	0.31 (13)
C8—C1—C5—C6	−177.12 (11)	O4—C12—C13—C8	176.95 (12)
O1—C1—C5—C4	35.38 (12)	C11—C12—C13—C8	−1.98 (19)
C8—C1—C5—C4	−70.15 (13)	O4—C12—C13—C2	0.3 (2)
O2—N1—C6—C7	−178.46 (12)	C11—C12—C13—C2	−178.59 (14)
O2—N1—C6—C5	2.48 (16)	O1—C2—C13—C8	31.49 (13)
C4—C5—C6—N1	−0.72 (15)	C3—C2—C13—C8	154.22 (12)
C1—C5—C6—N1	106.32 (14)	C4—C2—C13—C8	−72.49 (13)
C4—C5—C6—C7	−179.75 (13)	O1—C2—C13—C12	−151.53 (15)
C1—C5—C6—C7	−72.71 (17)	C3—C2—C13—C12	−28.8 (2)
O1—C1—C8—C9	151.77 (14)	C4—C2—C13—C12	104.49 (17)
C5—C1—C8—C9	−102.85 (16)		

### Hydrogen-bond geometry (Å, º)

**Table:**

*D*—H···*A*	*D*—H	H···*A*	*D*···*A*	*D*—H···*A*
C1—H1*A*···O2^i^	1.00	2.35	3.2928 (17)	156
C14—H14*C*···O2^ii^	0.98	2.59	3.5340 (19)	161

Symmetry codes: (i) *x*−1/2, −*y*+1/2, *z*−1/2; (ii) −*x*+1, −*y*+1, −*z*+1.
